# Early Depiction of a Parotid Tumour in the “School of Athens” (1509–1511) by Raphael (1483–1520)

**DOI:** 10.1007/s12105-017-0848-4

**Published:** 2017-09-01

**Authors:** Hutan Ashrafian

**Affiliations:** The Department of Surgery and Cancer, Imperial College London, St Mary’s Hospital, 10th Floor Queen Elizabeth the Queen Mother (QEQM) Building, Praed Street, London, W2 1NY UK

**Fig. 1:**
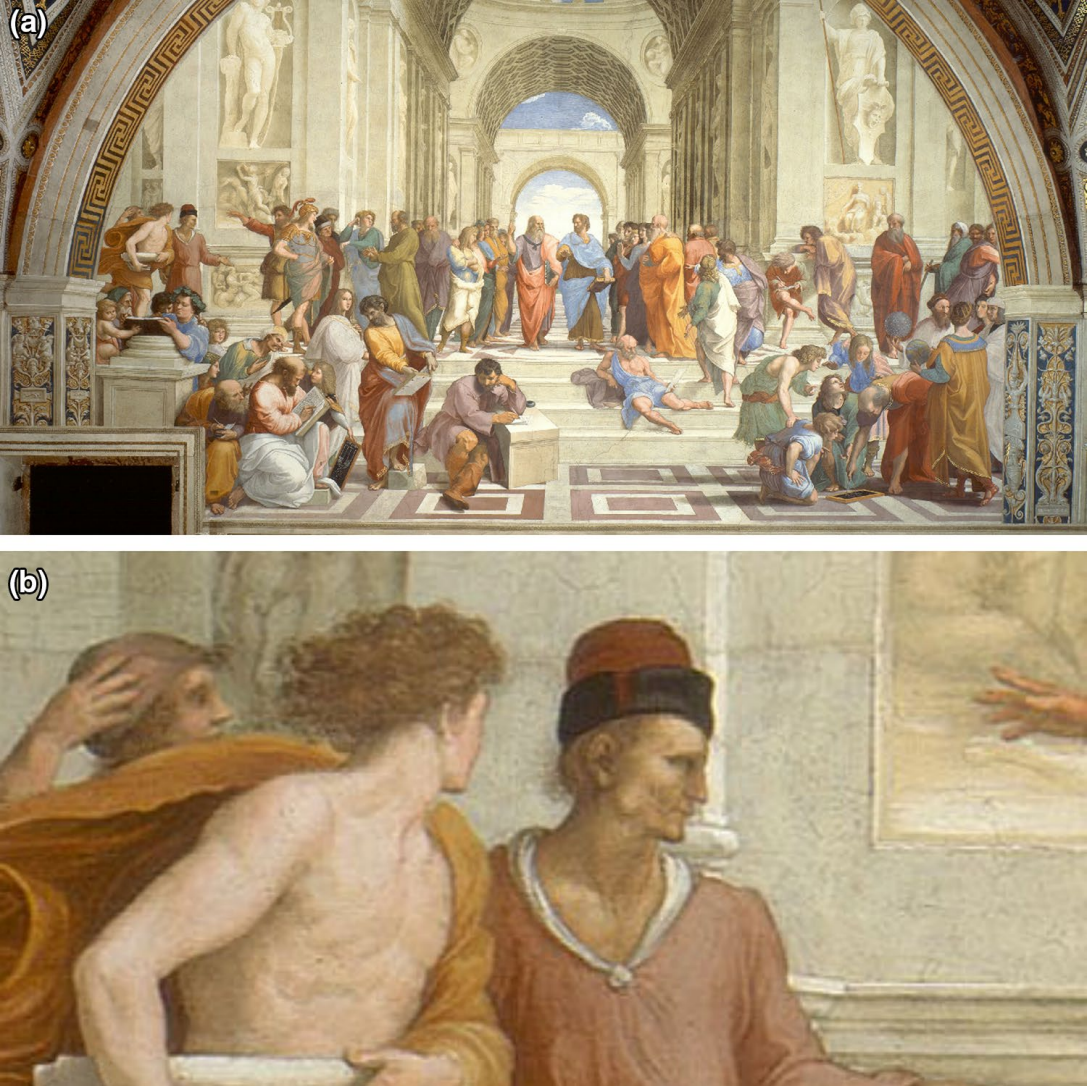
The School of Athens, Raphael (1509–1511), **a** complete painting **b** close-up of the face of an individual with a right parotid tumour © Apostolic Palace, Vatican City

The *School of Athens* of 1509–1511 (Fig. 1a) is considered amongst the greatest art works of the High Renaissance. It was painted by Raphael (Raffaello Sanzio da Urbino, 1483-1520), who is considered one of the ‘trinity’ of great masters from that period (alonside Leonardo da Vinci and Michaelangelo) and represents the pantheon of luminary philosophers and mathematicians.

On studying the painting, I note that a figure in the upper left area of philosophers demonstrates a clear right-sided parotid swelling or tumour (Fig. 1b). The identity of this individual is not well known. Whilst recently some sources have considered this individual to bear resemblance with the famed poet Dante (Durante degli Alighieri c. 1265–1321), all other portraits and sculptures of Dante lack evidence of any parotid swellings rendering the association between the two unlikely. The position of individual in the painting corresponds to quadrant of the painting considered to house logical and rhetoric philosophers, possibly some of the pre-Socratics. More likely the image represents the characteristics of a real-life model used by Raphael for this painting.

As the individual was painted during the renaissance, the common causes for a salivary gland tumour such as ionizing radiation and tobacco smoking exposure are unlikely here. Consequently, the differential for disease pathogenesis here include: infective causes (such as mumps, local parotitis or infections from distant sites), alcohol excess, underlying liver cirrhosis, neoplastic causes from other sites or familial neoplasms, sialoliths (salivary duct stones), Sarcoidosis and Sjögren syndrome. Other differential diagnoses include those for any lump such as a lipoma, abscess, sebaceous cyst, vascular malformation or lymphadenopathy.

In view of the age of this painting, this is likely the earliest depiction of a parotid tumour and serves to highlight the longstanding presence of salivary gland disease that is notable during the renaissance whilst additionally offering insight into the comprehension, method, origin and pathological associations of this prominent painting from a genius artist.

